# Raters’ perceptions of rating scales criteria and its effect on the process and outcome of their rating

**DOI:** 10.1186/s40468-022-00168-3

**Published:** 2022-08-01

**Authors:** Nasim Heidari, Nasim Ghanbari, Abbas Abbasi

**Affiliations:** Department of English Language and Literature, Faculty of Humanities, Persian Gulf University, Bushehr, 75169 Iran

**Keywords:** Raters, Perception, Writing assessment, Rating scale criteria, Analytic scale, Holistic scale

## Abstract

It is widely believed that human rating performance is influenced by an array of different factors. Among these, rater-related variables such as experience, language background, perceptions, and attitudes have been mentioned. One of the important rater-related factors is the way the raters interact with the rating scales. In particular, how raters perceive the components of the scales to further plan their scoring seems important. For this aim, the present study investigated the raters’ perceptions of the rating scales and their subsequent rating behaviors for two analytic and holistic rating scales. Hence, nine highly experienced raters were asked to verbalize their thoughts while rating student essays using IELTS holistic scale and the analytic scale of ESL Composition Profile. Upon analyzing the think-aloud protocols, four themes emerged. The findings showed that when rating holistically, the raters either referred to the holistic scale components to validate their ratings (validation) or had a pre-evaluation reading to rate in a more reliable way (dominancy). In analytic rating, on the other hand, the raters used a pre-evaluation scale reading in order to keep the components and their criteria to memory to evaluate the text more accurately (dominancy) or regularly moved between the text and the scale components to assign a score (oscillation). Furthermore, the results of a Wilcoxon signed-rank test showed that when using the holistic and analytic rating scales, the raters assigned significantly different scores to the texts. On the whole, the results revealed that the way the raters perceived the scale components will affect their judgement of the texts. The study also provides several implications for rater training programs and EFL writing assessment.

## Introduction

Writing assessment is a complicated process. The outcome of the writing assessment derives from an interaction among many elements of assessment including rater, test taker, scale, scoring, and practice. Therefore, the quality of rating has a significant bearing for the interpretation of outcomes (Ghanbari & Barati, 2020; Hamp-Lyons, 2007), and hence, different interactions regarding all these aspects of assessment can lead to different outcomes. In writing assessment, the impact of rating scale on the rating process (Barkaoui, 2010b), complexity of rating criteria (Joe et al., 2011), and raters’ perception can induce the lowest inter-rater reliability. On the other hand, an essay has lots of features that are the basis of rating; therefore, identifying the text components and bringing them in a conformity with the scale’s components is a demanding job. To increase the reliability of the assessment, raters should not misconceive the rating components as it negatively affects the consistency of their rating performance.

The evaluation of writing is a subjective process, and human raters predominantly rate the texts using rating scales which might affect the assessment practice. In fact, without having a clear picture of what happens in the raters’ cognitive process, it is impossible to claim whether their evaluations and subsequently the rating outcomes are at all fair or not (Cumming et al., 2002; Ghanbari & Barati, 2014). As a result, this shortage puts the scoring validity and consequential validity at risk (Weir, 2005). Different factors contribute to inconsistency in the rating process, among which raters’ perceptions and justifications of scoring decisions are more prominent. It is not expected that all raters perceive the rating scales’ components in a consistent and similar way. It is also inconceivable to gain the same results from all the raters. Most of the previous scale effect studies are restricted to comparing different types of scales (e.g., Bacha, 2001; Barkaoui, 2010b, 2011). Furthermore, despite doing numerous researches on rater behavior in the framework of performance assessment, few researchers have addressed the problem of rater’s perception in the process of rating in EFL contexts (Eckes, 2008). As long as assessment and rating are conducted by humans, they are inevitably influenced by personal beliefs, ideas, and preferences (Eckes, 2008). The components of the rating scale, the wording of the rubrics, the impressions brought with raters, and the potential interaction of these elements can affect the raters’ perceptions and, therefore, the scores they assign (Goodwin, 2016).

Due to the vagueness and lack of explicitness in the application of rating scales’ components, raters may not have an explicit perception during the rating process, and it may affect the outcome of rating. As a result, to gain a clear picture of the raters’ perceptions of rating scales and their effects on the rating process and outcome, in this study, the interaction between the raters’ perceptions and rating scales was investigated. The main concern pursued in this research is considering the raters’ perceptions of rating components regarding two different rating scales which might be significant in a number of ways: first, because there is a possibility that raters do not have the explicit perceptions of rating components, the misperceptions may decrease the reliability of the assessment. If the raters perceive the rating components precisely, this will help teachers and raters during the rating process to get a better outcome. Second, this explicitness also helps the teachers who evaluate students’ writings holistically and analytically through focusing on textual features in the writing class which lead to the development of students’ writing skill. In other words, a clear perception of the rating scales’ components can also help the raters focus on other writing features. Third, it seems that lack of explicit and precise perception of rating rubric leads to unfair scoring. If the error of measurement reduces through consistency of rating, accuracy and fairness of raters will increase. Fourth, according to Russikoff (1995), when raters rate ESL texts holistically, they pay more attention to language use despite the robust content and organization. Fifth, following Weigle (2002), in a holistic rating scale, descriptions of features are combined and thus make the score challenging to interpret; therefore, the findings of this study can help the raters have a robust interpretation of the test scores which further enhance the validity of the writing assessment.

### Literature review

Writing assessment is considered as one of the difficult tasks in language assessment (Coombe, 2010). The characteristics of raters in writing assessment are the idiosyncrasy that can be sought in the cognitive, attitudinal, and background variables. These factors are affected by raters’ appropriate education, training, and prior experience (Khodi, 2021; Kim & Lee, 2015; Rahayu, 2020). To prevent assigning inconsistent scores, concentrating properly and exclusively on evaluation of examinee’ writing performance during the rating process, and applying appropriate rating procedures, raters should receive professional training. However, although the knowledge of writing assessment and the efficacy of raters in scoring accuracy are the basic necessities, the correct perception in rating process can ascertain how fair and effective they rate a text (Meissel et al., 2017). This emphasizes different raters’ perceptions as a source of inconsistency in the rating process (Davidson et al., 2000).

Perceptions have been identified as idiosyncratic and subjective notions, views, and evaluations of someone’s performance which significantly contribute to second language teaching and learning because they form the base of teachers’, test takers’, and raters’ views, judgments, and interpretations (Brown, 2009). Ono et al. (2019) defined raters’ perceptions as “important aspect of raters’ scoring behaviors and views of (in) effective performance, as well as the challenges they face while scoring” (p.70). Barkaoui (2007) investigated the effects of rating scales on raters’ perceptions, outcomes, and scoring processes. Four raters rated 24 essays silently adopting a holistic scale and then two subsets of four essays applying a holistic scale while thinking aloud. The findings showed that in contrast to holistic scores, which were more reliable, there were inconsistencies in multi-trait scores which might be because of not training the raters. Among all aspects, the content and organization were of higher reliability than style, grammar, and mechanics. The essay organization is considered to be a broad concept referring to both physical aspects such as paragraphing, cohesion, and deep textual aspects such as coherence (Li & Huang, 2022**;** Liu & Huang, 2020). This feature of writing is taken into consideration as one essential component in all rating scales.

In an explanatory study on integrated written tasks, Ono et al. (2019) compared teacher raters using holistic and analytic scales for reliability and validity so as to unravel their perceptions of rating. The findings illustrated that raters encountered various challenges while scoring. According to Panadero and Jonsson (2020), as analytic scales focus on the components rather than the whole, the rating process may undergo the fragmentation. In another study, Eckes (2008) ascertained the extent of differentiation among the raters’ perceptions of criterion importance and examined the performance of the importance rating scale. Findings revealed that raters acted differently regarding the significance of the various criteria. Besides raters’ different perceptions, to some degree, rater background variables led to scoring profile differences. Humphry and Heldsinger (2019) investigated perceptions of assessment criteria related to identifying writing performance levels. Anchoring direct pairs of writings, raters determined the well-organized and proficient writing according to an analytic rating scale consisting of ten ordered criteria and a holistic scale. The results illustrated that raters considered some features such as ideas and text structure more prominent than others. Jeong (2019) made a comparison between the effects of a binary scale and an analytic scale on rating performance and scores while using two groups of participants: teacher raters and expert raters. He found that because the binary scale lessened the rater cognitive load and was easy to use, the raters were more consistent in their ratings. In a seminal work, Hijikata-Someya et al. (2015) examined to what extent the raters’ perceptions of native English-speaking and nonnative English teacher raters differ when rating a writing using the holistic scale. Findings illustrated that the former group of raters perceived the evaluation of two components, namely content and *language use* as difficult. In contrast, the non-native teacher raters considered rating challenging as associated with *vocabulary.* In a mixed-method research, Wang et al. (2017) investigated raters’ perceptions, and judgments assigned scores to integrated writings (reading to write task). Through analyses of raters’ perceptions, researchers found that there was an inconsistency in raters’ perceptions towards particular aspects of the texts.

### This study

The analysis of raters’ perceptions can reveal the critical features of the scale components and also identify how the raters behaved when facing the rating components. In this study, the aim was to identify and categorize the raters’ perceptions regarding the components of the rating scales. Raters always face various challenges to score accurately which may originate from different perceptions towards the scale components. This study sought for a better understanding of the interaction between the rater and the scales components and how it might affect the process and outcome of their rating. Hence, to achieve these goals, the following research questions were posed in this study:

- How do raters perceive the rating components when using holistic and analytic rating scales?

- Do different raters’ perceptions affect the rating outcome and process using holistic and
  analytic rating scales?

## Methodology

### Design of the study

In this study, a mixed-methods design was adopted. Both qualitative and quantitative data was collected to identify the raters’ perceptions regarding the rating scale components. The first phase was qualitative which focused on the data obtained from think-aloud protocols (TAPs). Because writing rating process is not easy to identify, a qualitative approach could assist the researcher in effectively interpreting the depth, variety, and complexity of raters’ thought processes (Creswell & Poth, 2018). In the second phase, the outcomes of the ratings were quantitatively analyzed in order to find out if rating using different rating scales would affect the score the raters assigned.

### Participants

The raters who participated in this study were nine experienced Iranian EFL teachers who were selected using convenience sampling from two Iranian state universities of Persian Gulf University (PGU) and Salman Farsi University of Kazerun (SFKU) located in the southwest of the country. These raters were teaching the undergraduate courses of advanced writing and essay writing. They were experienced writing teachers with at least 5 years of teaching and assessing writing experience in the university. The age range of the participants was between 35 and 59 years old. The research participants were chosen from both female and male teachers (five males and four females). Four out of five male raters had MA degrees, and the remaining one had PhD. In addition, three out of four female raters hold PhD, and the remaining one had MA. Also, only three out of nine raters had participated in a workshop related to rating writing assessment. All the 9 raters agreed to contribute to the study. They were also assured that their data would be confidential and would be used only for research purposes.

### Instruments

In order to collect data for the present study, six instruments were used including writing tasks, two rating scales including IELTS holistic scale and ESL Composition Profile (Jacobs et al., 1981), training manual, demographic information sheet, and think-aloud protocols (TAPs). The following briefly explains each.

#### Writing task

*The writing texts used in this study were randomly selected from a body of thirty essays written under exam-like conditions by EFL undergraduate students. As the final exam of the writing course, the students were asked to write an essay (about 160 words) on the topic “how do you recover emotionally from the disasters”. The essay was used for two rounds of rating and scoring holistically and analytically. The raters were next asked to rate the essays based on the holistic and analytic rating scales they were provided with.*

#### IELTS holistic scale

*IELTS holistic rating scale was used in the study. Based on this scale, the text is given a score from 1 to 9.* One of the attributes of this scale is its global approach in rating and assigning. The holistic scoring takes the whole writing into account as the product and assigns a score to the overall writing quality. One of the advantages of holistic scale can be its cost-effectiveness which makes it an appropriate instrument for the large-scale evaluation of written practices, particularly for placement decisions (Ghalib & A-Hattami, 2015).

#### ESL Composition Profile

ESL Composition Profile (Jacobs et al., 1981) as an analytic rating scale was used in this study. The reason behind selecting this instrument was Sasaki and Hirose (1999) who claimed that Jacobs et al.’s (1981) ESL Composition Profile was a commonly used scale and one of the most preliminary well-designed rubrics to assess L2 writing though not without its pitfalls. The rubric consists of five main components of a written text: content and organization which refer to the prepositional content, unity, cohesion, and coherence of the text; vocabulary refers to the range, lexical diversity, word choice, and word form mastery; language use focuses on grammatical points, accuracy, and syntax; and last is mechanics which refers to paragraphing, capitalization, and generally superficial aspects of a text. Each component is rated according to four levels ranging from very poor to very excellent. The scores for each level in each component differ.

#### Demographic information sheet

In order to obtain the background information of the raters, they asked them to fill in a demographic information sheet containing a personal profile, current professional status, educational history, and the experience of assessing and teaching writing.

#### Training manual

Training manual consisted of an instruction sheet which the raters received in order to guide them to conduct TAPs in a consistent way. In addition, along with this manual, the researchers sent a recorded message in which they explained the think-aloud procedure precisely and provided some examples in order to clarify and adequately convey information about how to perform TAPs and also apply holistic and analytic rating scales.

#### Think-aloud protocols (TAPs)

In general, to capture essay rating processes and map out the raters’ mental processes when rating, the participants performed TAPs. The procedure involved verbal expression of their mental processes. For this, the raters were trained to verbalize their thoughts while rating an essay. The raters’ verbalizations were recorded to be analyzed next. The TAP technique originated from cognitive psychology which discovered the relationship between thoughts and words. In initial research, it was known as productive thinking and a means to see into the minds of individuals. Johnstone et al. (2006) claimed that “Because all cognitive processes travel through short-term memory and TAPs employ thoughts in individuals’ short-term memory, the conscious thoughts of the participants can be reported at the time they are processed”. TAPs focus on the understanding of the relationship between thoughts and words (Charters, 2003). As its name reveals, the purpose of the protocol is to “give the researcher insights into the processes of working memory” (Charters, 2003, p.70).

### Data collection procedure

Early in the study, a group of qualified EFL writing instructors were contacted to participate in this study. Due to the difficult situation caused by the COVID-19 pandemic, many teachers were reluctant to participate in face-to-face sessions. Therefore, a package including two rating scales, a training manual which consisted of an instruction of TAP along with a voice recorder, writing texts, and demographic information sheet was emailed to the teacher raters. Data collection proceeded in four stages: first, a personal demographic information was given to the raters to obtain such information as age, gender, experience of teaching, and rating writing. Second, a training manual was given to the raters in order to make them familiar with the TAP procedure. Third, they were asked to play the recorded voice which was attached to the manual to clarify how to do TAPs and also rate with the two scales as well. It is also worth mentioning that the raters were free to use either mother tongue (Persian) or English in performing TAPs. Fourth, the TAPs were carried out in order to see into the raters’ minds and investigate their thought processes during the rating process. Therefore, the raters were asked to verbalize their thoughts as they were rating the essay. They used two different rating scales. After rating using a particular scale finished, the easy received the score, and immediately rating with the next scale started. The raters also verbalized their thoughts while rating the essays. On average, the process took about 45 min for each rater. Then, the recorded TAPs were transcribed in order to codify their utterances and identify some patterns among them.

### Data analysis

In order to analyze the raters’ perceptions of the scales’ components during the rating process, the rating behaviors of nine raters obtained from TAPs were transcribed and then codified to seek for the general patterns emerging from the data. Categorical qualitative content analysis was used to analyze data. The analysis proceeded in four stages as below.

In order to familiarize with the data, the researcher reads through the transcripts before breaking down them into segments and meaningful units. The identification of each meaningful unit labeled with a code was the second stage. However, due to the nature of inductive coding, the codes changed as the study made progresses, and coding process was repeated several times. Additionally, it was checked whether all aspects of the content of transcriptions were covered regarding the purpose of the study. The third stage was categorization. In this stage, the codes were condensed, meaning that fewer words were used without missing content of the meaningful unit which led to the identification of some themes. The fourth stage was compilation, when the emerged themes were further refined. Moreover, to answer the second research question, using SPSS program, Wilcoxon signed-ranked test was used. This test was selected to compare two sets of scores which come from the same participants.

## Results

### Investigating the first research question

To answer the first research question, a focused analysis was conducted to show how the raters perceive the rating components when using holistic and analytic rating scales. It is worth mentioning that out of the nine raters, three misfit raters were identified, meaning that they did not use the scales’ components for rating the texts.

### Raters’ perception of holistic scale

Upon the analysis of the raters’ interactions with the holistic scale components, two major themes of validation and dominancy emerged. With regard to the validation theme, the raters rated the text impressionistically and then assigned the score. Next, to validate their scoring, they referred to the scale and read the band descriptors of the scores given. Reading of the band descriptors persuaded them to revise the score. Regarding the dominancy theme, the raters scanned the scale before beginning to rate so that they could review the band descriptors and develop a master plan of their evaluation. In what follows, the stages the raters went through in their rating processes for both themes are delineated (Table 1).

**Table 1:** Raters’ interactions with components of holistic scale

Theme 1: Validation	
1. Self-monitoring focus	
2. Assigning score	
3. Referring to scale to read the band descriptors	
4. Revision	
5. Verification	
Theme 2: Dominancy	
1. Pre-evaluation scale reading	
2. Detailed rating	
3. Consulting scale to reread band descriptors	
4. Assigning score	
5. Verification	

#### Validation

This theme can be defined in the way that raters attended to the rating scale in order to validate their scoring. According to Table 1 above, two raters (R1and R6) applied this theme in their rating. Both raters explicitly mentioned that their rating would be based on the holistic scale. For example, rater 1 mentioned that “scoring the composition based on the holistic method, ok! I will read the text first and then I will have my overall assessment of the passage”.

Initially, both raters articulated a general impression of the essay. Their meticulous evaluation based on self-monitoring focus assured them to assign score before reading the scale’s band descriptors. Obviously, the score given was in accord with their initial impression. To validate their scoring, they referred to scale to read the components of the score given. Reading the band score’s components caused them to revise the score subsequently giving evidence from the text. As it is shown below, reading the band descriptors of the assigned score led to the revision of that score, and as a result, the rater selected a different score:

R1: I think (pause) I think hum the score for this text falls in um (pause) 6 band which says the test taker has an effective command of the language despite some inaccuracies, inappropriate usage and misunderstandings. He/she can use and understand fairly complex language, particularly in familiar situations. No! I think I give it 7, because the vocabulary the writer has used is appropriate and… pause…. I give it 7.

The above quotes illustrate how by using the scale’s components the raters made or revised their scoring decisions. After revising the score, the raters provided evidence from the text to justify their assigned score. These raters did not read other band scores’ components. They only read the band descriptors which they thought was appropriate for the essay and, after reading the components, selected another one which was only one band higher or lower than the initial score. It seems that the raters were already familiar with the band scores’ components and were able to make an approximate estimate of the score.

#### Dominancy

To define the theme, it should be said that the raters scanned the scale before starting the evaluation process in order to dominate the scale. The reason was to get a general picture of the whole scale and adequately dominate the scale band descriptors during the rating process. For example, raters 2 and 3 clearly stated that they had a pre-evaluation reading:

R2: I prefer to read this scale before correcting the writing; however, you explained it explicitly, just for reviewing, …um nine bands, you now it is general focusing on definite guidelines, expert users say…

After rating the text accurately, the raters consulted the scale to assign score. The band scores and their components were reviewed so that this whole reading of band scores could help them reach the appropriate score. Rereading the band descriptors, analyzing them, and giving evidence from the text led them to pay attention to different aspects of writing and employ a larger and more varied number of the essay-rating criteria. Interestingly, R2, R3, and R9 who had a pre-evaluation of the scale read almost all of the band descriptors. Among all these four raters, only R4 did not read the whole band scores’ components. She returned to scale to only read one band descriptor which was in accord with her initial impression. As evidence, the way rater 3 interacted with the text and the scale comes below:

R3: …she was not an expert user! Actually, she was not very good user, in fact, good user: The test taker has operational command of the language, though with occasional inaccuracies, inappropriate usage and misunderstandings (long pause) well! I saw some complex language and most of sentences were clear. Competent user: The test taker has …. modest user: The test taker has a partial command of the language and copes with overall meaning in most situations, although is likely to make many mistakes. He/she should be able to handle basic communication in own field. Of course, I think that the vocabulary she used was in advanced level (pause)...but little knowledge on content and organization of writing….

Overall, for both themes, the raters focused more on strengths of the essay using the scale components. Also, content and organization among all the scale components received more attention. Moreover, when the raters encountered the difficulties, they used resolution strategies such as anchoring the holistic scale with other scales or comparing two band descriptors or using analytic rating scale criteria while adopting the holistic rating. Here is an example of anchoring band two descriptors by R3: “The writer has lots of problems related to the complexity. This band deals with the use of complex language, while the previous one focuses on communication.”

### Raters’ perception of analytic scale

The analysis of the raters’ TAPs showed that in the analytic rating, the raters frequently channeled their attention to details. All the raters expressed their initial impression of the essay through looking at the layout, cross-outs, following rating the essay precisely. However, their rating and scoring processes were based on the scale components. In addition, their interactions and behaviors were fundamentally different. Generally, raters who interacted with the scale components applied two themes of dominancy and oscillation in their rating processes*.* Regarding the dominancy, the raters had a pre-evaluation scale reading in order to keep the components and their criteria to memory; therefore, they could evaluate the text more accurately. For the oscillation theme, the raters regularly moved between the text and the scale components. In this section, both themes are elaborated as below (Table 2):

**Table 2:** Raters’ interactions with components of analytic scale

Theme 1: Dominancy	
1. Pre-evaluation scale reading	
2. Appraising the text	
3. Consulting the scale	
4. Re-reading components criteria	
5. Assigning score	
Theme 2: Oscillation	
1. Continuous move between the text and the scale	
2. Reading component criteria	
3. Assigning score	

#### Dominancy

The raters read the whole scale before their rating so that they could dominate over the scale. Here, the raters (R1, R2, R3, R9) scanned the analytic rating scale (Jacobs et al., 1981) prior to their evaluation in order to include the components into their rating and take into account the multifaceted nature of writing aspects. As an example, R3 emphasized, “I have not used this scale for a long time, it’s better to take a look at it, five components, yes, content, um, so, knowledgeable, substantive, development of thesis…”. Additionally, this pre-evaluation scale reading was done because they wanted to review the components and the criteria of each component. Then all four raters precisely rated the text and consulted the scale to articulate score decisions according to the components:

R1: ok this is the paragraph, let me read for the details and the grammatical (pause) lexical, organization. (pause) and the rest of the components of the analytical scale….

As shown below, rater 2 reads the criteria of all levels in each component reversely in order to reach an appropriate level, interpreted and translated them into Persian, and commented on the mistakes of the text according to those criteria so that he could select one of the levels as the final decision.

R2: In language use, again, at first, I read the last level which is very poor: virtually no master of sentence, …., no, the communication can be seen in writing some complex sentences, for example, the second line of the first paragraph did not have lots of errors, fair to poor: major problems in simple and complex construction, …, as I told before the problem of dangling which is related to agreement, I select 21-18 for language use.

Rater 1 reads only the criteria of one level for each component in agreement with his general impression which indicated that he was impartially certain about that score because after reading the criteria, he did not revise the score. Rater 3 reads only two levels of each component. Rater 9 reads the criteria of first level (excellent to very good) in each component as the definition of that component and then selected one level out of three regarding her general impression to read its criteria. When the rater wanted to assign score for one of the components (mechanics), she was dubious about that component which resulted in referring to the text and rating the component again.

#### Oscillation

This theme can be defined in the way that the raters constantly oscillated between the text and the components of the scale. In other words, following the oscillation theme, raters (R4 and R6) moved continuously between the text and the scale. The raters had a separate evaluation for each component in detail and then read the criteria of that component to articulate the score.

R4: In terms of organization, what I see here is that excellent to very good: fluent expression, actually the first paragraph has been written partly well , some errors are seen here for example the ideas have not developed there I consider fair to poor : non fluent, ideas disconnected, ….ideas are clearly stated, uhmm no, …well, good to average : somewhat choppy, loosely organized… yes as I see ideas are not connected, limited support, logical but incomplete…, lots of irrelevant details, good to average is reasonable…..in terms of vocabulary….

As shown above, this style of rating components could not help the raters in decision-making process since they had simultaneous conflicting reaction towards each component. In other words, they showed ambivalence in assigning score even after several times reading the criteria and giving evidence from the text. Therefore, the scale components did not provide adequate and explicit information for the various aspects of the text and might block the interpretations of the raters.

R6: In terms of vocabulary, I choose very poor because completely describes the essay: Essentially translation. Actually she has little knowledge of this topic, no word related to topic, not idiom, of course now I’m seeing that in the last part of this level it has said: Not enough to evaluate, so let me read another one fair to poor: Limited range, frequent errors of word form, idiom form…um, I think it’s ok, I assign this score to this component… um let me return to text I see she has used look for in wrong way, lead to, um , cause or for example damaged buildings leave no place for people to live without worrying their lives.. no, I select the previous one very poor because look for was translated.

Similar to rater 9 in the dominancy theme, R6 forgot to attend to mechanics and hence referred to the essay to scan the text and look for the errors of mechanics:

R6: Uhmm, mechanics, very poor: No mastery of conventions, dominated by errors of spelling, punctuation, capitalization you know I did not look at the text carefully to see whether the writer observed punctuation, capitalization (pause) I see that there is a comma after however, poor handwriting, Frequent errors of spelling, punctuation, capitalization, paragraphing, poor handwriting, right, 3 is satisfying.

In fact, although the raters used the same scale to justify their scores, they adopted different approaches in their evaluation. For instance, some raters disregarded a component in rating process, and when they wanted to mark in scoring process, they were obliged to consult the text in order to re-rate that component. While R6 and R9 returned the text to assess the mechanics, R6 usually resorted to the strategy of comparing two levels of each component to settle down her problem about the vagueness of scale wording. Moreover, more detailed and cautious evaluation involved iterative rating and scoring behaviors with too much of a cognitive load. That is why when raters were not able to reach a sound decision on yielding score for each component, they tend to interpret the criteria meticulously or compare the criteria of two levels or two scales. According to the dominancy, pre-rating scan of the scale helped raters select a level out of four more easily and master the scale’ components. This group of raters, in their consultation of the scale, tried to re-read most of the levels’ criteria. On the other hand, in oscillation, the raters were doubtful in selecting one level and assigning score for that component.

### Investigating the second research question

The second research question aimed to examine whether the raters’ perceptions of the two scale components influence the rating outcome. To answer this question, Wilcoxson signed-ranked test was used to examine the possible differences between the scores assigned by using the analytic and holistic scales.

The results of the descriptive statistics on the two scales are shown in Table 3. In holistic scale, scores were scored out of 9, while in analytic scale, they were calculated from the total score of 100 (Table 4).

**Table 3:** Descriptive statistics of holistic and analytic scales scores in this study

Descriptive statistics					
	*N*	Mean	Std. deviation	Minimum	Maximum
Holistic	9	5.44	1.424	4	7
Analytic	9	62.11	13.606	40	76

**Table 4:** Wilcoxon signed-rank test for the two scales

Test statistics^**a**^	
	Analytic — holistic
*Z*	−2.666b
Asymp. sig. (2-tailed)	.008
a. Wilcoxon signed-rank test	
b. Based on negative ranks	

As the table above shows, the significance level is less than 0.05 (*p* < 0.05) which indicates that there was a difference between the raters’ scores assigned holistically and analytically. In other words, there was a significant difference between the mean of the two sets of scores meaning that the raters assigned significantly different scores to the text using the holistic and analytic rating scales. Therefore, it was found that having different perceptions on the rating scales’ components and the way the raters interacted with them affected the final rating outcome assigned.

## Discussion

The analysis of TAPs showed that the raters perceived the analytic and holistic scales’ components in different ways. Regarding the first research question, the most striking results to emerge from the data are that the raters referred to the scales’ components prior to their scoring, after looking for mistakes, before and after assigning the score holistically and after scoring each component of the analytic scale separately. It was also found that three raters followed dominancy in their both holistic and analytic ratings. They adhered to the guidelines of scales and preferred to rate the texts considering all the components of the scales.

All in all, fifty rating behaviors were coded in the present study. The raters in the analytic rating mode showed a wider range of these rating interactions and complicated thought processes probably because they had not used the scale increasingly, or because the scale components did not cover all the possibilities, the raters tended to formulate various strategies to help them deal with problematic dimensions of the rating process (Lumley, 2002) such as ambivalence, disregard of components, and complexity of the scale wording.

Simple application of scales does not certify an effective evaluation, and an ill-designed rating scale might even block understanding of criteria included in the scale. On the other hand, as Panadero and Jonsson (2020) claimed, rating scales are scoring guidelines that contain described components at different levels of standards. As mentioned before, for both themes, the raters had some difficulties with the scale components which could have been derived from lack of familiarity with the application of the scale or complicated nature of the scale. These strategies included anchoring, translation, much scrutiny of the rating components, reading reversely, and individual inference making about the scale components. Moreover, the content of the analytic scale increases the working memory of the raters (Hirai & Koizumi, 2013) because of having a wide range of criteria. Thanks to this, it might be difficult for raters to analyze and interpret the scale components, and through adopting those strategies, they lessened the cognitive load. On the other hand, findings of Jeong (2019) showed that the scale design has a greater effect on the raters. Thus, designing rating scales and the rating criteria should provide an explicit and reliable foundation for scoring judgments, over and above distinguishing writing performance levels (Weigle, 2002). This type of rating scale (Jacobs et al., 1981) used in this study has been criticized for the vagueness of the components’ criteria. Turner and Upshur (2002) criticized the criteria for being irrelevant to the test task and its context, and the criteria are improperly grouped at descriptor levels. An example can further clarify this observation. Two of the raters expressed their ambivalence in assigning score to all analytic scale components. According to the observations, raters felt more confusion in their ratings which implied that they got it quite demanding to clearly select one level. This finding is in line with Jeong (2019) who concluded that raters had to consider multiple areas in the analytic rating process and experienced more hesitation and rating conflict. Therefore, these strategies like anchoring happened more in analytic evaluations which can be due to the vague wording of the scale.

Moreover, none of the raters felt ambivalent in scoring holistically which indicated that raters when scoring holistically are fundamentally in the center of their own cognitive process since they need to decide on one general score and not focus on several separate scores. Therefore, it can be attributed to the conciseness of holistic rating scale’s components which help raters in the decision-making process. The raters frequently preferred to read the band scores of the holistic rating scale and components of analytic rating scale in the sequence in which they appeared which agreed with the findings of Winke and Lim (2015). Another finding of this study is that in their holistic rating, it took the raters a long time to read, rate, and look for mistakes in the text because as Barkaoui (2010a) explained, raters need time to rationalize their score. In analytic evaluations, on the other hand, the raters rated the text faster but had difficulty making quick decisions when applying the components. Fast decision-making hinges on how much attention raters paid to which components.

Moreover, variations in rating processes are frequently attributed to individual differences in the raters’ attentional focus (Cumming, 1990; Eckes, 2008). In this study, the raters put their attentional focus on all aspects of writing when rating the essay holistically but did not consider most of them in scoring process in order to justify the assigned score and only judged based on the essential features like organization and content. This is in good agreement with Huot (1990) reports of a study on the rating process in which the experienced raters applied a holistic rating scale. The findings showed that the raters were highly influenced by content and organization. Findings from Plakans *and* Gebril (2017) and Winke *and* Lim (2015) also showed that ignoring organization component in the test taker’s writing enhances the scoring variability and lessen the reliability of scoring ESL/EFL writing. Additionally, in the analytic scoring, the raters were in agreement with two components of organization and language use. This finding supported the results of Barkaoui (2010b) who asserted that raters attached more importance to organization when they applied analytic scale to rate the EFL writings. Besides, the findings lend support to Winke and Lim (2015) who reported that raters put high attention on organization component which led to the highest consistency. Li and He (2015) also mentioned that the organization received the most attention while rating EFL essays analytically.

To add more, it was also found that in the analytic rating scale, the raters adhered to the scale’s components even when they wanted to give evidence, whereas in the holistic rating, they mixed their own criteria with the content of the scale or at least focused on two components: organization and content. Noticeably, this finding was also in line with those by Barkaoui (2007) and Li and He (2015) who reported that the application of the analytic scale resulted in less frequent use of self-made criteria. In addition, in comparison with the holistic scale, the raters had a tendency to confine their attention to just those on the scale while rating analytically (Goulden, 1994). In fact, the order of the components appearing in the analytic rating scale (Jacobs et al., 1981) does not reflect the importance of the components. In a study, Winke and Lim (2015) claimed that because content and organization are arranged first in the scale, raters put more attention to these components. In this study, it was found that the raters focused slightly less on the mechanics component in the analytic rating; therefore, two raters consulted the essay to re-rate the component while reading that component’s criteria. During the essay rating process, they might not examine mechanic errors or might not bear them in mind. This lack of consideration might stem from primacy effect (Underwood, 1975). The primacy effect brings a tendency to those raters to ignore the mechanics component because this is listed last in the analytic scale, and the raters may well perceive it as the least important dimension. This finding is in line with the Winke and Lim (2015) who claimed that mechanics component received less attention among all analytic components.

Regarding the second research question, the results of Wilcoxon signed-rank test showed that there was a difference between the raters’ scores assigned holistically and analytically. Mumford and Attay (2021) characterized the factors that lead to discrepancy among raters’ scores: any rater types, rating scales, interpretation of rating scales, and the factors that make a text complicated to score. Different perceptions and interactions towards the scale’ components can lead to the discrepancy as well. In other words, variations in rating behaviors constantly were observed which affected the score that the raters assigned to the essay. Anchoring, ambivalence, disregard of components, and individual inference making about the components were some differences in the raters’ perceptions. In this study, it was also found that the raters’ outcomes in the analytic scoring were more lenient than holistic scores even though in the former raters fluctuated across five components and a wide range of sub-components. On the other hand, Deygers et al. (2018) found individually distinctive di*ff*erences in rater severity in both analytic and holistic scoring. In addition, the findings of the present study match well with Holzknecht et al. (2018) who reported signi*fi*cant disagreement on the analytic and holistic rating scores. Eckes (2008) claimed that inconsistency among equally trained raters is usually derived from their individual differences in their way of reading essay and considering the scale components they perceive as important. As a result, differences in the raters’ scoring behaviors might bring about low inter-rater agreement. However, Lumley (2002) explained that different scoring behaviors do not fundamentally result in inconsistency among the raters. In this study, although the raters were equally trained to apply rating scales components, an inter-rater agreement was not observed among them which can further support previous findings in Choi (2002) that training does not necessarily affect the inter-rater reliability.

## Conclusions

In sum, the findings of the present study showed that the raters are predominately obsessed with content and organization when using the holistic scale. In other words, in holistic rating, the raters mostly focused on the content and organization components, while in the analytic rating, the raters almost considered all aspects of the text such as length, layout, and capitalization. In addition, in holistic rating, the raters emphasized on the strengths of the essay, whereas in the analytic scoring, the raters put all their attention on the weaknesses of the text as well. Moreover, the findings revealed that rating scale components assist raters to take into account all aspects of writing during the scoring process. Overall, the findings showed that EFL writing raters will possibly be affected to various and unpredictable extents by scales components, writing criteria, and different interpretations that might have applied before to rate essays and frequently through their long experience. Thus, by making these components, interpretations, and criteria more examinable, more valid results will be obtained.

Applying the behaviors identified in this study in rater training programs can help them inform their own beliefs and priorities regarding the different types of rating scale components and act effectively as well in rating the texts. Knowing the particular way that the raters perceive the rating task can improve the quality of rating and lead to increasing the assessment accuracy and reliability of the scoring. The detailed interactions of the rater can also help identify the strategies raters adopt in their ratings. In this study, some problems in the rating process emerged which were due to the complexity and vagueness of criteria in the analytic components which in turn led to adopting translation, comparison, and rereading of criteria in each component. In order to improve scoring consistency and efficiency and promote assessment transparency, the complexities related to the concepts and descriptors of the components should be solved and considered in training programs. In this way, the rater can do the rating task in a consistent way. Another implication of the study concerns the values that rating scale developers attributed to different components of a scale. The results of the qualitative data on the scales in the rating process showed how the raters weighed the scale components such as content, organization, and mechanics. In fact, getting access to such list of priorities will assist training program organizers to be aware of how to develop the programs in a way that inform the raters to cover and better understand similar weight of all rating components of analytic and holistic scales in the best possible way.

This study also suffers from a number of limitations. This study was carried out with a small sample size (i.e., nine raters and one essay) in the particular EFL context of Iran. However, investigating these research questions with larger samples and in different contexts might create different findings. According to Barkaoui (2010a), TAPs do not reflect and show a whole picture of rating process and affect both rating process and outcomes as some raters mentioned in their demographic information. Therefore, these intrinsic limitations of TAPs can affect the validity of the findings. According to Shohamy et al. (1992), training plays an influential part in affecting raters’ behaviors using rating scales to rate the text, particularly through clarification of rating criteria. Due to the limitations caused by COVID-19 pandemic, many raters were reluctant to participate in face-to-face sessions. Therefore, think-aloud sessions were conducted in a remote way which could affect the validity of the TAPs obtained.

Further studies can be conducted to identify the raters’ perceptions regarding the structurally similar scales. Addressing the process of rating holistically and analytically in two time intervals is also required in order to shift the focus from the identification of the raters’ perceptions to the comparison of raters’ perceptions regarding the scales components. This study considered the rating of only one sample essay. Future investigations can survey the identification of raters’ different perceptions on more essays. According to Charters (2003), a combination of interviews and TAPs would provide a more detailed picture of the participants’ thought processes. Based on Qi (1998), a follow-up interview might permit the participants to validate the interpretation or clarification of think-aloud protocols.

## Data Availability

The data associated with this study would be available upon request.

## Abbreviations

IELTS: International English Language Testing System
ESL: English as a second language
EFL: English as a foreign language
SFKU: Salman Farsi University of Kazerun
TAPs: Think-aloud protocols
PGU: Persian Gulf University
